# Script Concordance Tests: Guidelines for Construction

**DOI:** 10.1186/1472-6947-8-18

**Published:** 2008-05-06

**Authors:** Jean Paul Fournier, Anne Demeester, Bernard Charlin

**Affiliations:** 1 Département de Pédagogie Médicale, Faculté de Médecine de Nice Sophia Antipolis, France; 2 École Régionale de Sages-femmes, Faculté de Médecine de Marseille, France; 3 CPASS, direction de la recherche, Faculté de Médecine, Université de Montréal, CP 6128, Succursale centre-ville, Montréal, Québec, H3C 3J7, Canada

## Abstract

**Background:**

SCT is used to assess clinical reasoning in ambiguous or uncertain situations. It allows testing on real-life situations that are not adequately measured with current tests. It probes the multiple judgments that are made in the clinical reasoning process. Scoring reflects the degree of concordance of these judgments to those of a panel of reference experts.

**Method:**

SCT is an item format that is gaining acceptance in education in the health professions. However, there are no detailed guidelines on item writing, test scoring or test optimization.

**Results:**

The item format is described and the steps for preparing and administering reliable and valid SCTs are presented.

**Conclusion:**

SCTs probe examinees on a specific clinical reasoning task: data interpretation, a crucial step in the clinical reasoning process. It is inferred that a high degree of concordance corresponds to optimal use of information in the context of these specific tasks and therefore provides an indication of clinical reasoning quality.

## Background

According to script theory [1-3], clinicians mobilize networks of organized knowledge, called "scripts", to process information and progress toward solutions to clinical problems. For example an ear, nose and throat specialist working with an outpatient suffering from vertigo is focusing on his or her knowledge of vertigo-inducing illnesses. As soon as a new patient comes into the room, complaining of a cervical mass for instance, the vertigo knowledge is "washed out" and networks of knowledge related to cervical masses are called to mind with direct questions to ask, physical exams to do or investigation/treatment options to decide on. These knowledge networks are acquired during clinical training and refined with each clinical encounter [3]. They are specifically adapted to the tasks clinicians commonly perform.

According to theory [3], scripts are made up of links between illnesses, clinical features and management options. Health professionals progress toward solutions to clinical problems with hypotheses (or management options) and their related knowledge networks (scripts) in mind. They actively use them to constantly make judgments on the effect that each new piece of information has on the status of the hypothesis or option [3]. Script concordance testing (SCT) is based on the principle that the multiple judgments made in these clinical reasoning processes can be probed and their concordance with those of a panel of reference experts can be measured. This provides a tool for assessing clinical reasoning [4].

The test format is used to assess reasoning in ambiguous or uncertain situations. These situations frequently occur in daily practice, especially for primary care physicians [5,6]. They are nevertheless poorly measured with usual tests. Clinicians find the test appealing because its cognitive tasks are the same as those they carry out constantly in their daily practice. A series of studies looking at fields such as family medicine, midwifery, surgery or radiology [4,7-11] have documented the reliability and construct validity of test scores. This paper specifically addresses the need for a description of item writing and rules governing the preparation and administration of reliable and valid SCTs. It describes the specific features of SCT and reiterates the general rules to follow in constructing SCTs or any other educational tests.

## Test principles

**Figure 1:**
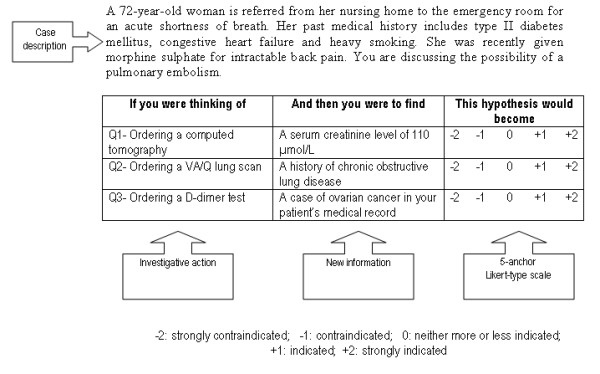
Example of case from the investigation section of an SCT.

The test is case-based. Cases, described as short scenarios, always incorporate uncertainty. Several options are relevant to solve the diagnostic or management problem posed by the situation. A case, with its related questions, constitutes an item (Figure 1). Scenarios are followed by a series of questions, presented in three parts. The first part ("if you were thinking of") contains a relevant diagnostic or management option. The second part ("and then you were to find") presents a new clinical finding, such as a physical sign, a pre-existing condition, an imaging study or a laboratory test result. The third part ("this option would become") is a five-point Likert scale that captures examinees' decisions. The task for examinees is to decide what effect the new finding has on the status of the option, in direction (positive, negative or neutral) and intensity. This effect is captured with a Likert scale because script theory assumes that clinical reasoning is composed of a series of qualitative judgments [3].

## Test construction

For any evaluation [12], the first step of test construction is to determine the basic purpose of the evaluation. Is it to initiate a learning process – for instance in professional development – or to assess learning achievement at the end of educational activities? For which level is it intended: students, residents or practising health professionals? All these questions influence the construction of the test. Some principles taken from classical test theory [13] are applicable to SCT construction. For example, a test seeking to discriminate among examinees should be composed of questions with medium difficulty (so that the variance of examinees' scores will be maximized). On the other hand, a diagnostic test, used to identify areas of specific weakness for low-ability students, must contain a substantial number of questions which are relatively easy for the general population of examinees [13].

An important issue, for any test, is content validity, i.e. the extent to which the test samples or "covers" the area of clinical reasoning under consideration. This issue is often addressed using a specification table: all areas of the field are specified and cases are chosen so as to ensure adequate sampling. For instance, in an emergency medicine test aimed at residents, situations were selected by three experts with respect to their frequency (e.g. congestive heart failure or chronic obstructive pulmonary disease), their severity (e.g. shock, torn aorta) and to the patients' sex ratio and ages [14]. Test developers can then construct items in a way that allows examiners to conclude that performance with respect to these items represents the performance that would be displayed for the entire field.

## Test material production

Clinical scenarios and their related questions can be written by a single person, but teams of two test writers optimize creativity and productivity. Casual observation shows that with larger groups, lengthy and non-productive discussions on content, wording or trivial details occur. Instructions for test writers may be: 1- Identify a series of clinical situations you have recently encountered in your clinical practice, not necessarily complex or unusual, but containing uncertainty (the situation must call for at least two diagnoses or management options). 2- What are the relevant hypotheses or options for these situations? 3- What data would you look for in these situations to help you progress toward the solution?

This phase produces material made up of cases, options and data. Questions related to each case are written using a combination of options and data while keeping several issues in mind. The first is to focus on key features [15], i.e. on data – positive or negative – that are useful in progressing toward a solution. The second is the need to spread answers over each anchor of the Likert scale (if most of the questions are on -1 or +1, test-wise students will quickly identify this bias). The third is that Likert scale anchors must be clearly defined to prevent any ambiguity. The fourth is the meaning of the zero anchor on the scale, which relates to data that have neither a positive nor a negative impact on the option status. It is not an easy task for a novice to affirm that a particular piece of data has no significance in a given context. This requires experience. The 0 anchor is not a shelter for candidates without a clear opinion, unlike the anchor "I don't know" in the Likert scale of an opinion poll.

## The place of uncertainty

SCTs deal with uncertainty at two levels: 1- within the case depicted in the scenario (by design, this level of uncertainty is always present); 2- in questions nested within cases: some may contain uncertainty and some may not, since reasoning on the significance of data in clinical contexts may sometimes induce different interpretations among clinicians and sometimes provide a clear answer. Questions for which most answers on the panel are minus or plus 2 are often questions that provide clear answers.

With more traditional assessment tools, such as multiple choice questions (MCQs), a question that induces discrepancies in the answers given by a reference panel is considered to be of poor quality, while it has been shown that SCT questions leading to variability within the panel better detect levels of clinical experience in a group of examinees [8]. Questions with consensus (low variability) among panel members have less discriminative power, while questions with large margins of disagreement (high variability) reflect measurement error (noise) and are not useful. Nevertheless, experience shows that it is useful for SCTs to include questions on which panel members agree (low variability questions). These items assess knowledge of well-established solutions to well-defined problems. This kind of item is close to the rich-context, multiple-choice question, but the item format and the task required of examinees are both different.

## Cases

Cases, described as short scenarios, present challenging clinical situations in a few sentences. Even experts cannot provide one single solution to the problem, either because not all the data are available (e.g. diagnosis or management issues), or because several attitudes or options are justifiable (e.g. therapeutic issues), or because there is no consensus in the literature on the strategy to use. The scenario sometimes ends with a sentence describing the problem to be solved, as shown in Figure 1 (an investigation item), but most of the time the problem is implicit. While SCT is commonly used in diagnosis, treatment, or management issues, it could be used in more particular aspects such as ethical or professionalism issues.

## Item format

It is important to address the following issues.

1- Likert scale anchor descriptors differ according to the types of questions asked: diagnostic, investigation, treatment, or management. Table 1 suggests specific anchor descriptors for these different tasks. Because of this change of anchor descriptors, a single case generally comprises questions of the same type, while other cases will explore other kinds of clinical reasoning tasks.

**Table 1:** Descriptors suggested for 5-anchor scales aimed at measuring diagnosis, investigation and treatment tasks.

If you were thinking of the following diagnosis...	...and the following new information were to become available...	...this hypothesis would become...
Diagnosis option	New information	-2 -1 0 +1 +2
-2: very unlikely -1: unlikely	0: neither likely nor unlikely	+1: more likely +2: very likely
**Anchor descriptors for the diagnosis format**		

If you were considering the usefulness of the following investigation...	...and the following new information were to become available...	...you would then consider the investigation...
Investigative option	New information	-2 -1 0 +1 +2
-2: useless -1: less useful	0: neither more nor less useful	+1: useful +2: very useful

**Anchor descriptors for the investigation format (utility issue)**		

If you were considering the risk-benefit ratio of the following investigation...	...and the following new information were to become available...	...this new information would make the investigation...
Investigative option	New information	-2 -1 0 +1 +2
-2: strongly contraindicated -1: contraindicated	0: neither more or less indicated	+1: indicated +2: strongly indicated

**Anchor descriptors for the investigation format (risk-benefit issue)**		

If you were considering the utility of the following treatment...	...and the following new information were to become available...	...you would then consider this treatment...
Treatment option	New information	-2 -1 0 +1 +2
-2: useless -1: less useful	0: neither more or less useful	+1: useful +2: very useful

**Anchor descriptors for the treatment format (utility issue)**		

If you were considering the risk-benefit ratio of the following treatment...	...and the following new information were to become available...	...you would then consider the treatment...
Treatment option	New information	-2 -1 0 +1 +2
-2: strongly contraindicated -1: contraindicated	0: neither more or less indicated	+1: indicated +2: strongly indicated

**Anchor descriptors for the treatment format (risk-benefit issue)**		

2- If two successive questions address the same options, examinees may think that any new clinical information provided by the two questions is cumulative. To prevent this, it is recommended to alternate options between questions (for instance, in Figure 1, question 1 may deal with ordering a computed tomography, question 2 with ordering a VA/Q lung scan and question 3 with ordering a D-dimer test). This notion of independence of information in each question should be clearly explained by the test instructions.

3- Pursuing a case with a succession of developing scenarios probing diagnosis, followed by investigation or treatment (cascade testing), is not recommended. It is better to have a new case for each item. If not, several items become interdependent, which violates the principles of test construction [13].

## How many cases, how many questions?

What is the optimal number of cases and questions within cases to maximize the reliability of test scores? Case specificity means that the success of any case is specific to that case [17,18]. Successfully solving one problem is a poor predictor of whether an individual will be able to successfully solve another problem. Thus, to assess experience in a given field, it is necessary to sample situations broadly.

Nevertheless, experience shows that tests done on many cases with just one question per case are too cognitively demanding for examinees [9]. Generalizability D studies indicate that using fewer cases, with an average of 3 questions per case, improves reliability [19]. Tests comprising 20 cases and 60 questions, for one hour of testing time, reach Cronbach coefficient alpha values higher than 0.75 [4-10]. It is therefore advisable to ask several questions for each case, as long as those questions address critical or essential elements.

## Likert scale

The structure of the scale is the same for the whole test with, for instance, negative values on the left, 0 in the neutral position and positive values on the right. Scales should be one-dimensional in order to avoid ambiguity and measurement errors. The use of wording such as "contraindicated" and "indicated" in investigation or treatment formats, allows examiners to reinforce the uncertainty issue by introducing a legal or risk-benefit issue (Table 1). The item in Figure 1 is a good illustration. "Contraindicated" or "Strongly contraindicated" refers to the potential contrast medium-induced kidney damage in an elderly patient who previously experienced slightly impaired renal function. On the other hand, "indicated" or "strongly indicated" refers to the potential benefit of the computed tomography in confirming or ruling out the suspected pulmonary embolism [20]. In this perspective, the item accurately captures the cost-benefit approach of decision-making in an uncertain context, which closely resembles real life [8].

How many anchors should SCT Likert scales have? Theoretically, a scale should be as wide as possible to collect as much information as possible, but at a certain point examinees no longer know for sure if they should provide such and such an answer, and this produces noise rather than information in measurement. Initial SCT studies were composed of seven-anchor Likert scales. It quickly became evident that this was not beneficial, and five-anchor scales are now generally used. Continuing medical education, where SCT is used as a learning stimulus, is an exception [21]. In this setting, participants are asked to complete an SCT individually, discuss with other participants in small groups to reach a common answer, then compare that answer with those of experts, to initiate the learning process. In this situation it appears that using three-anchor scales is more effective at inducing educationally relevant discussions.

## Scoring

SCT scoring involves comparing answers provided by examinees with those of a reference panel composed of physicians with experience in the field being assessed. Panel members are asked to complete the test individually, and their answers are used to develop the scoring key [16]. Credits for each question are derived from the answers given by the reference panel.

For each answer, the credit is the number of members that chose that answer, divided by the modal value for the question. If, for a given question, fifteen panel members chose "-2," two chose "-1" and one choses "0" credit for the "-2" is 1 (15/15), credit for the "-1" is 0.13 (2/15), and credit for the "0" is 0.06 (1/15). For the non-chosen options, "+1" and "+2," the credit is 0. With this method, all questions have the same maximum (1) and minimum (0) value. Scores obtained on each question are added to obtain a total score for the test. This number is then divided by the number of questions and multiplied by 100 to get a percentage score.

The aggregate scoring method described above is the most commonly used method [4]. However, it is important to acknowledge that the optimal SCT scoring method is still debated [22]. The aggregate method has many implications for implementing a classical theory test model, and much psychometric research remains to be done on level of consensus, score scale and the relationship with a discrimination index, among others. Also, validity research is required to understand the relationship of SCT with more traditional knowledge item formats, performance assessments and clinical reasoning.

## Panel size, composition and recruitment

Gagnon [23] has shown that, for high stake examinations, 15 panel members are required in order to obtain acceptable reliability estimates (Cronbach's alpha coefficient). The values of these estimates rise with larger panels, but with more than 20 members, improvement is only marginal. For lower stake examinations, for instance formative assessment within a clinical rotation, smaller panels can be used. However, panels with less than 10 persons are associated with more error in reliability estimates.

Composition is another important issue. The basic idea behind SCT is to compare students' or residents' performance with a group of persons who are legitimate representatives of the profession (or the specialty) to which they wish to belong. Therefore, panels should be made up of physicians with good overall clinical experience in the field rather than experts from narrow parts of the field. Panel composition also depends on the assessment goal. If, for instance, one wishes to assess family physicians' clinical knowledge of gynecology, should the panel consist exclusively of family physicians with a gynecology practice or gynecology specialists? The answer depends on the test developers' goals.

Considering how difficult it often is to recruit members of an examination jury, the need to recruit 15 to 20 members for a reference panel may be a concern. In fact, the SCT actually presents an advantage over other test formats in that panel members are asked to answer questions that are very similar to those they ask themselves in their own clinical work. Furthermore, as opposed to many other tests that require preparatory review for optimal performance, a clinician can fill out the test at any time without any preparation. These two reasons explain why, in practice, it is not difficult to recruit members for panels of reference. Nevertheless, panel member anonymity is required. SCT is not intended to provide individual scores on the experts' performance.

## Test optimization

SCT psychometric qualities are enhanced by careful quality control at all test construction steps. Clear instructions to item writers are necessary, and the quality of items produced may be checked using tools such as the grid [24] presented in Table 2. The first version of a test should be reviewed by a small group of experienced physicians from the field and of persons whose skill and knowledge levels are similar to those of the examinees. Reviewers are asked to verify the quality of the wording and the relevance of questions. Low-quality questions or cases are discarded or rewritten. Because the test format is unusual for most examinees, tests should begin with an explanatory introduction and a few sample items for practice and familiarization.

**Table 2:** Script Concordance Test item quality grid, adapted from Caire

Scenario	• Describes a challenging situation, even for experts	yes	no
	• Describes an appropriate situation for examinees tested	yes	no
	• The scenario is necessary in order to understand the question and to set the context	yes	no
	• The clinical presentation is typical	yes	no
	• The scenario is correctly written	yes	no
Questions	• Questions are developed following a key-feature approach	yes	no
	• In the experts' opinion, the options are relevant	yes	no
	• The same option is not found in two consecutive questions	yes	no
	• The new information (2nd column) makes it possible to test the link between the new information and the option (1st column) in the described context	yes	no
	• Likert scale anchors are clearly defined and unambiguous	yes	no
	• Questions are developed to spread the answers equally over all the values of the Likert scale	yes	no
	• Questions are developed to provide balance between low and high variability	yes	no

Experts' panel	• Number between 10 and 20	yes	no
	• The experts' panel includes experienced physicians whose presence in a jury is appropriate to the level of the examinees assessed	yes	no
	• Experts take the test individually, in exactly the same conditions as the examinees	yes	no

Once the test has been taken by a group of participants, it can be optimized by item analysis [13]. Coefficients of difficulty, discrimination and impact on the test's overall reliability are computed at question and case levels. With item analysis, shorter and more reliable tests can be produced.

**Figure 2:**
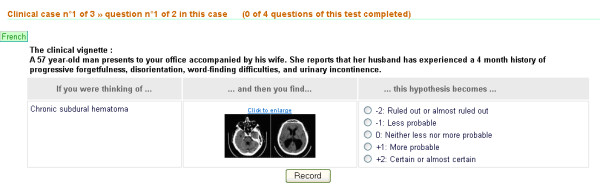
Example of test item administered on line.

While tests can be paper-based, on-line testing allows simulated situations to be enriched with images (dermatology, radiology) or videos (endoscopies, neurology signs). On-line testing also facilitates test administration, scoring and presentation of results to examinees. An example of on-line testing [26] can be seen in the Figure 2.

## Conclusion

With SCT, examinees are probed on a specific clinical reasoning task: data interpretation, a crucial step within the clinical reasoning process [25] Scores reflect the degree of concordance with decisions made by a panel of experienced physicians. It is inferred that a high degree of concordance corresponds to optimal use of information in the context of these specific tasks and therefore provides an indication of clinical reasoning quality. Several studies showing SCT capacity to discriminate among examinees of different levels of experience [4,7,11] provide evidence in favour of construct validity.

SCT does not replace other clinical competence assessment tools such as OSCEs or rich-context MCQs. It complements them in strategies for assessing comprehensive clinical reasoning. Its format allows examiners to explore a facet of clinical reasoning that is usually excluded from traditional medical assessments but frequently faced in daily clinical practice: reasoning in situations for which there are no clear correct answers.

## Competing interests

The authors declare that they have no competing interests.

## Authors' contributions

JPF and BC have conceived the paper and made the first draft. AD has contributed on critical elements of the test format and context of use. All authors have read and approved the final manuscript.

## Pre-publication history

The pre-publication history for this paper can be accessed here:


